# Editorial: Suicidal behavior and depression among perinatal women: research, prevention, intervention, and treatment

**DOI:** 10.3389/fpsyt.2024.1491809

**Published:** 2024-09-20

**Authors:** Sami Hamdan, Yari Gvion, Vita Postuvan

**Affiliations:** ^1^ Academic College Tel Aviv-Jaffa, Tel Aviv Jaffa, Israel; ^2^ Bar-Ilan University, Ramat Gan, Tel Aviv, Israel; ^3^ University of Primorska, Koper, Slovenia

**Keywords:** postpartum depression (PPD), suicidal behavior, risk factors, vulnerable populations, perinatal mental health care, interventions

The perinatal period, including pregnancy and the first year after childbirth, is often a time of joy and new beginnings. Despite that, for many women, this period also brings significant psychological challenges. Depression and suicidal behaviors among perinatal women are serious issues that affect mothers and their children as well as the family. The studies in this Research Topic dive deep into these challenges by exploring risk factors, causes, and potential interventions that can help women navigate this difficult period more safely and effectively.

## Unpacking the risk factors

One of the main themes resulting from the research is the identification of risk factors that contribute to postpartum depression (PPD) and suicidal behaviors. Studies in this Research Topic have examined how different physical and psychological factors can increase a woman’s risk. For instance, higher body weight, a history of mental health issues, including major depression, and certain socioeconomic factors have been found to raise the likelihood of developing PPD (Wedajo et al.; Yu et al.). On the other hand, factors like being older at the time of the first birth and having more years of education seem to offer some buffer against these mental health difficulties (Zuo et al.).

Interestingly, another study examined the association between Gut health and postpartum depression (Zhang et al.). The results suggest that the composition of gut bacteria might play a role in either heightening or reducing the risk of PPD and open up possibilities for new treatments that target gut health as a way to help prevent or manage postpartum depression.

## Exploring effective interventions

Timing and effective interventions are another major focus of the Research Topic. One study from India (Szajna et al.) highlights a community-based approach in which health workers deliver a simple, acceptable, and effective method of reducing depression symptoms. It is a good example of how low-cost, scalable interventions can make a significant difference, especially in resource-limited settings.

Another study focused on the role of exercise in preventing depression during pregnancy (Liu et al.). The results confirm that regular physical activity plays a vital role in reducing depressive symptoms, especially when started early and maintained throughout the pregnancy. These results reinforce the idea that including exercise in prenatal care routines is needed to support the mental health of expectant mothers.

## Addressing challenges in vulnerable populations

Different studies in this Research Topic address the specific mental health challenges among vulnerable populations, including adolescent mothers and women with autoimmune diseases. For instance, Miafo et al. reported on the high rates of mental disorders and suicidal risk among young mothers. These findings highlight the urgent need to make mental health services accessible to adolescent mothers, especially in areas where these challenges are most common.

Another study examined the association between postpartum depression and autoimmune diseases (Yu et al.), indicating that PPD may raise the risk of developing conditions such as type 1 diabetes and Hashimoto’s thyroiditis. These findings emphasize the need for comprehensive monitoring of both mental and physical health among postpartum women to help prevent long-term complications.

## Improving screening and support systems

The need for better screening and support systems is another key theme. One study examined the effectiveness of different screening questions for detecting suicidal risk among perinatal women (Dudeney et al.). The findings show that many women were uncomfortable with direct language about suicide, which poses challenges for healthcare providers in identifying those at risk. The results underscore the urgent need for more sensitive and culturally appropriate screening tools to ensure no woman slips through the cracks.

A study conducted in Ethiopia on self-harm among postnatal mothers attending immunization clinics highlights the prevalence of this issue and its associated risk factors (Wedajo et al.). The authors suggested that healthcare providers need to be more attentive in recognizing and addressing the signs of self-harm to improve the overall health outcomes for these women.

## Moving forward

This Research Topic provides a comprehensive look at the complexities of perinatal depression and suicidality. From understanding the underlying risk factors to exploring effective interventions, these studies highlight the challenges and the potential for significant improvement in perinatal mental health. As we continue to learn from these studies, there is a promising future where we can build better support systems for women during the perinatal period and reduce the incidence of these serious mental health issues.

To move further, research in the future might need to broaden the horizons and include risk factors beyond the individual. Much needs to be done to understand the social and environmental factors and thus include interventions that do not only address the needs of the group with the highest mental health needs but rather rely on public health approaches. The perinatal period brings along many normative yet manageable changes if we have appropriate and broad support available, including psychological, relational, social, and related systems. This highlights the need for further research to address these gaps in knowledge and guide future interventions and policies in the field of perinatal mental health.

We would like to thank all the contributors to this Research Topic for their valuable insights and dedication to improving our understanding of the mental health challenges in perinatal women. We also encourage continued collaboration and innovation in this field.

